# Smarter vaccine design will circumvent regulatory T cell-mediated evasion in chronic HIV and HCV infection

**DOI:** 10.3389/fmicb.2014.00502

**Published:** 2014-10-06

**Authors:** Leonard Moise, Frances Terry, Andres H. Gutierrez, Ryan Tassone, Phyllis Losikoff, Stephen H. Gregory, Chris Bailey-Kellogg, William D. Martin, Anne S. De Groot

**Affiliations:** ^1^EpiVax, Inc., Providence, RI, USA; ^2^Institute for Immunology and Informatics, University of Rhode Island, Providence, RI, USA; ^3^Department of Medicine, Rhode Island Hospital and the Warren Alpert Medical School at Brown University, Providence, RI, USA; ^4^Department of Computer Science, Dartmouth College, Hanover, NH, USA

**Keywords:** HIV, HCV, T cell epitope, immunoinformatics, vaccines, cross-reactivity, regulatory T cells, MHC class II

## Abstract

Despite years of research, vaccines against HIV and HCV are not yet available, due largely to effective viral immunoevasive mechanisms. A novel escape mechanism observed in viruses that cause chronic infection is suppression of viral-specific effector CD4^+^ and CD8^+^ T cells by stimulating regulatory T cells (Tregs) educated on host sequences during tolerance induction. Viral class II MHC epitopes that share a T cell receptor (TCR)-face with host epitopes may activate Tregs capable of suppressing protective responses. We designed an immunoinformatic algorithm, JanusMatrix, to identify such epitopes and discovered that among human-host viruses, chronic viruses appear more human-like than viruses that cause acute infection. Furthermore, an HCV epitope that activates Tregs in chronically infected patients, but not clearers, shares a TCR-face with numerous human sequences. To boost weak CD4^+^ T cell responses associated with persistent infection, vaccines for HIV and HCV must circumvent potential Treg activation that can handicap efficacy. Epitope-driven approaches to vaccine design that involve careful consideration of the T cell subsets primed during immunization will advance HIV and HCV vaccine development.

## THE CHALLENGE

Despite modern advances in preventing disease by vaccination, persistent viral infections continue to pose a major challenge to vaccine development. The most prominent examples are HIV-1 and HCV infections, which remain two of the largest global public health challenges today. Highly effective medications are now available, but these are still inaccessible to the majority of at-risk individuals mainly due to their cost, and limitations on access to healthcare in the developing world countries where HIV and HCV are most prevalent. Unfortunately, no vaccine candidate against AIDS or hepatitis C is currently nearing market approval. Only a handful of HIV vaccine efficacy trials have been completed, and none have yet been completed for HCV (Honegger et al., 2014). While a range of strategies to treat infection and prevent transmission have been studied and implemented, it is widely believed that an effective vaccine for these global health threats is essential to stopping new infections worldwide.

Development of effective HIV and HCV vaccines is lagging because traditional strategies for developing vaccines have failed to overcome the ability of the viruses to evade the human immune response. Many obstacles to vaccine development have been uncovered: (i) extensive viral genetic diversity, enabling HIV and HCV to evade humoral as well as cell-mediated immune responses (von Hahn et al., 2007; Haaland et al., 2013), (ii) lack of suitable animal models, (iii) establishment of latent reservoirs following integration into the host genome soon after infection (in the case of HIV; Perreau et al., 2013), and (iv) lack of clear correlates of protective immunity. We recently hypothesized that viruses that tend to cause chronic diseases *mutate their T cell epitopes toward greater homology with the human genome*. The existence of several highly homologous T cell epitopes, some of which induce regulatory T cell (Treg) responses, has been confirmed. We postulate that this is an important means by which HIV and HCV evade effective T cell responses, and that failure to account for such epitopes may have contributed to the failure of certain vaccine approaches undertaken to date. Methods for discovering HIV and HCV Treg-activating epitopes and strategies for improving HIV and HCV vaccines are described briefly here.

## VIRUSES FIND NEW MEANS TO EVADE HUMAN IMMUNE RESPONSES

No matter where one stands on the subject of the correlates of immunity to HIV and HCV infection, it is generally acknowledged that strong and broadly reactive HIV- and HCV-specific CD4^+^ T cell responses are required for control of acute viral infections (Rosenberg et al., 1997; Gerlach et al., 1999). Early collapse of the CD4^+^ T cell response impairs antibody production and CD8^+^ T cell responses; thus, an effective vaccine needs to induce long-lived CD4^+^ T cells capable of sustaining these essential components of immunity (Lichterfeld et al., 2004; Schulze Zur Wiesch et al., 2012).

CD4^+^ T cells are activated by virus-specific epitopes presented in the context of class II MHC by antigen presenting cells. Identification of class II MHC epitopes has been an active area of research for characterization of antigen-specific HCV and HIV CD4^+^ T cell responses in infection and vaccination and for construction of epitope-driven vaccines (De Groot et al., 2004; El-Awady et al., 2013; Karpenko et al., 2014; Mishra et al., 2014; Takei et al., 2014). Long-standing criteria for characterizing class II MHC epitopes include allele coverage in the human population and virus coverage among circulating strains, measured by how well an epitope represents a virus-induced response in the human host. Our group has published and validated methods by which this might be accomplished (De Groot et al., 2004; Koita et al., 2006; Mishra et al., 2014).

A significant epitope property that is beginning to gain wider attention is homology with host sequences. Viral epitopes with substantial homology to self are, at best, inert because of clonal deletion in the development of central tolerance; at worst, they may activate Tregs that suppress protective inflammatory responses and thereby enable viral persistence (Rolland et al., 2007; Frankild et al., 2008; Calis et al., 2012). It is well established that HCV-induced Treg activation is associated with extended chronic infections (Losikoff et al., 2012). Several studies of chronic HCV subjects have shown increased frequencies of natural CD4^+^ Tregs that express high levels of CD25, produce IL-10 (Cabrera et al., 2004), TGF-β (Bolacchi et al., 2006), and FoxP3 (Li et al., 2007), and suppress IFN-γ production (Sugimoto et al., 2003) and proliferation of HCV-specific CD8^+^ T cells (Boettler et al., 2005; Rushbrook et al., 2005). In the case of HIV, the role of Tregs in infection is currently being debated and requires further investigation to identify Treg subsets that may be responsible for suppressing non-specific T cell activation (beneficial) and of HIV-specific effector T cell responses (detrimental; Chevalier and Weiss, 2013).

We believe that vaccines for HIV and HCV must account for potential Treg activation that can diminish efficacy, particularly when a strong CD4^+^ T helper immune response is required. Ideally, vaccines should be carefully designed to reduce or eliminate potential Treg-activating sequences. That is possible today with the availability of immunoinformatic tools to predict class II MHC epitopes that may stimulate Tregs.

For over 20 years and until very recently, T cell epitope-mapping algorithms have focused on the MHC-facing side of epitopes and ignored the T cell receptor (TCR) face; thus, their usefulness was limited to identifying MHC ligands. While MHC binding is necessary to stimulate a T cell response, it is not sufficient. Hence, immunoinformatic-identified MHC ligands have been screened experimentally for T cell activation to validate predictions. Because Tregs are responsive to HIV and HCV epitopes, T cell assays should be performed to ascertain which T cell subsets are activated. Indeed, Treg-activating epitopes have been discovered using overlapping peptide arrays and tetramers in HCV core, NS3, NS4, and NS5 antigens (Li et al., 2007, 2009; Ebinuma et al., 2008; Langhans et al., 2010) and HIV Gag (Angin et al., 2012). These approaches are extremely cumbersome, however, when screens are conducted on a genomic scale with the intent to broadly cover human MHC diversity.

## ACCELERATING THE DISCOVERY OF VIRAL CAMOUFLAGE SEQUENCES

An informatic tool that rapidly screens thousands of candidate epitopes could address the problem of viral immune escape, but also needs to consider the vast variability and degeneracy of TCRs, making prediction extremely challenging. Fortunately, the problem can be significantly reduced by searching directly for virus-encoded human homologs that potentially stimulate natural and inducible Tregs, even if some Treg-activating epitopes are not necessarily human homologs. Specifically, pathogen sequences that bind MHC and share the same TCR-face with human MHC ligands may stimulate pre-existing Tregs that emerged from development of central and peripheral tolerance. Shared sequence patterns on the TCR-face are easily searchable.

To better define and rapidly assess viral camouflage epitopes, i.e., those epitopes homologous to human, we developed the JanusMatrix algorithm, which leverages our existing algorithm (EpiMatrix) to define MHC-binding peptide epitopes while searching for cross-conservation at the TCR-face. JanusMatrix can be applied to any viral or bacterial target protein to compare its TCR-faces to others in any genomes of interest. JanusMatrix analyzes the two faces of peptide sequences of pathogen origin for T cell activation potential (Moise et al., 2013). MHC-facing residues are analyzed for MHC binding potential using the Epi-Matrix epitope-mapping algorithm (De Groot et al., 1997). We have examined TCR-facing residues for conservation against a variety of sequence databases, including the complete human proteome, the human microbiome, and human pathogens (Moise et al., 2013). For example, we screened a wide range of human-host viruses for TCR-face similarity to self and discovered that chronic viruses generally appear more human-like than viruses that cause acute infection (He et al., 2014), and that H7N9 influenza may evade immune response in a similar way (De Groot et al., 2013, 2014).

**FIGURE 1:**
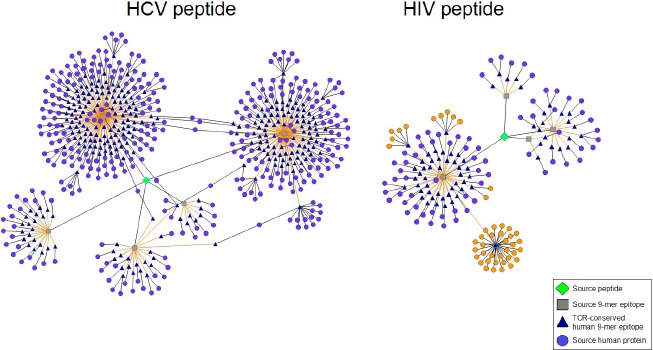
**Predicted Treg-activating HIV and HCV sequences possess TCR faces shared by numerous human proteins.** Epitope networks are shown, illustrating the abundance of TCR faces one HCV and one HIV peptide share with the human genome as determined by JanusMatrix analysis. The HIV and HCV source peptides are represented by green diamonds, their constituent 9-mer epitopes by gray squares, their cross-conserved partners in the human genome by blue triangles, and the source human proteins by light purple circles. In the HIV peptide (right) a single cross-conserved epitope can be found in 32 different HLA class I alleles; several additional 9-mer epitopes are cross-conserved with 12 other HLA sequences (source protein orange highlight).

Using JanusMatrix, we discovered a promiscuous class II epitope located within non-structural HCV protein p7 that exhibits homology with hundreds of human sequences (Figure 1). The epitope induces an increase in CD4^+^CD25^+^FoxP3^+^ Treg number and function in peripheral blood leukocyte cultures derived from an HLA-diverse cohort of HCV-infected patients, but not in cultures derived from patients who spontaneously cleared HCV or from non-infected individuals (Losikoff et al., 2014). A human analog of the HCV epitope stimulates Tregs in both HCV-infected and non-infected people, suggesting that tolerance to HCV is promoted by activating Tregs that recognize a common TCR-face. It is well known that HCV and HIV CD8^+^ and CD4^+^ T cell epitopes mutate over the course of infection, decreasing MHC binding as a mechanism of viral escape (Harcourt et al., 1998; Norris et al., 2006; Petrovic et al., 2012). The p7 HCV epitope exhibits a novel escape mechanism, evolving a TCR-face similar to that found in autologous T cell epitopes, thus stimulating Treg responses and suppressing immune clearance.

At first glimpse, JanusMatrix analysis indicates that HIV also exhibits curious patterns of potential T cell cross-reactivity. While searching HIV envelope sequences for TCR-face conservation in the human genome, we recently uncovered a high frequency of human MHC (HLA) molecule sequences that share a TCR-face with a highly conserved epitope located in the HIV envelope protein (orange circles, Figure 1). Because HLA is highly variable in the human population, conservation of this sequence across HLA subtypes is noteworthy. The phenotype of T cells responding to this epitope has not yet been evaluated in our laboratory but an Immune Epitope Database search identified a closely related epitope capable of stimulating CD4^+^ T cell recognition and proliferation (Atassi and Atassi, 1992). If these CD4^+^ T cells are T-effector in nature, their activation could restrict HIV expansion (Sanjuán et al., 2013). We believe that it is more likely, however, that T cells that recognize this epitope possess a Treg phenotype, which may promote HIV expansion and/or persistence instead.

These HIV and HCV epitope examples are consistent with our previously published observation that “hit-and-stay” viruses escape protective immune responses by stimulating cross-reactive Tregs (He et al., 2014). This suggests that Treg-activating HCV and HIV sequences may affect HCV and HIV vaccine efficacy. We find similar patterns of cross reactivity in EBV, CMV, and HSV, all viruses that establish chronic infection and for which no vaccine exists.

## ADDRESSING VIRAL CAMOUFLAGE IN VACCINE DESIGN

Homology with the human genome represents a novel means by which viruses that seek to establish chronic infections escape human immunity and ensure their survival. Better classification of viral epitopes as either effector-or Treg-activating will improve the design of HIV and HCV vaccines. Knowledge of which epitopes to include or exclude makes it possible to generate virus-specific T cell responses that are essential for protection and that sidestep suppression. One potential solution to the challenge of HCV and HIV vaccine design is to develop epitope-driven subunit vaccines, either as whole antigen protein vaccines, using a structure-based approach, or alternatively, as platform-neutral epitope-based vaccines that do not contain Treg-activating epitopes. Such vaccines would have major advantages over conventional, but as yet unsuccessful approaches because they would simultaneously account for viral and human diversity for the purpose of broad reactivity and promote protective virus-specific T cell responses.

Importantly, the impact of Treg-activating epitopes may have different inhibitory effects depending on the level of viral replication and immune activation in acute or chronic HIV infection. Thus, Treg epitopes to exclude may differ for prophylactic and therapeutic HIV vaccines. With respect to HCV, we believe the maximum effectiveness of an HCV vaccine used either prophylac-tically or therapeutically would be achieved by always excluding Treg-activating epitopes because (i) unlike HIV, which infects and replicates in Tregs, HCV is primarily hepatotropic and (ii) a better understanding of Treg function in chronic HCV infection could lead to treatments that are capable of balancing the competing needs for sustained effector T cell-mediated immunity and limited tissue damage moderated by Tregs (Self et al., 2013).

Finally, we are exploring means to fine-tune the epitope content of HIV and HCV vaccines to induce nuanced T cell responses associated with protection. We believe that careful design is needed to improve efficacy. Cross-reactivity at the individual level owing to HLA and HIV or HCV sequence variation may necessitate the development of personalized vaccines that contain T effector, but not Treg epitopes. While personalized vaccines may seem futuristic, tools are available to design such vaccines. As this technology becomes ever more accessible, there will be an even greater incentive to define the means of personalizing vaccines.

### Conflict of Interest Statement

Several of the coauthors on this manuscript are employees of EpiVax (Anne S. De Groot, Frances Terry, Leonard Moise, William D. Martin). Anne S. De Groot and William D. Martin are majority stockholders and Leonard Moise holds stock options. These authors recognize the presence of a potential conflict of interest and affirm that the information represented in this paper is original and unbiased observations.
